# Integrating fMRI and SNP data for biomarker identification for schizophrenia with a sparse representation based variable selection method

**DOI:** 10.1186/1755-8794-6-S3-S2

**Published:** 2013-11-11

**Authors:** Hongbao Cao, Junbo Duan, Dongdong Lin, Vince Calhoun, Yu-Ping Wang

**Affiliations:** 1 Unit on Statistical Genomics, NIMH/NIH, Bethesda, MD, USA; 2 Department of Biomedical Engineering, Tulane University, New Orleans, LA, USA; 3 Department of Biostatistics & Bioinformatics, Tulane University, New Orleans, LA, USA; 4 The Mind Research Network, Albuquerque, NM, USA; 5 Department of Electrical and Computer Engineering at the University of New Mexico, both in Albuquerque, NM, USA

## Abstract

**Background:**

In recent years, both single-nucleotide polymorphism (SNP) array and functional magnetic resonance imaging (fMRI) have been widely used for the study of schizophrenia (SCZ). In addition, a few studies have been reported integrating both SNPs data and fMRI data for comprehensive analysis.

**Methods:**

In this study, a novel sparse representation based variable selection (SRVS) method has been proposed and tested on a simulation data set to demonstrate its multi-resolution properties. Then the SRVS method was applied to an integrative analysis of two different SCZ data sets, a Single-nucleotide polymorphism (SNP) data set and a functional resonance imaging (fMRI) data set, including 92 cases and 116 controls. Biomarkers for the disease were identified and validated with a multivariate classification approach followed by a leave one out (LOO) cross-validation. Then we compared the results with that of a previously reported sparse representation based feature selection method.

**Results:**

Results showed that biomarkers from our proposed SRVS method gave significantly higher classification accuracy in discriminating SCZ patients from healthy controls than that of the previous reported sparse representation method. Furthermore, using biomarkers from both data sets led to better classification accuracy than using single type of biomarkers, which suggests the advantage of integrative analysis of different types of data.

**Conclusions:**

The proposed SRVS algorithm is effective in identifying significant biomarkers for complicated disease as SCZ. Integrating different types of data (e.g. SNP and fMRI data) may identify complementary biomarkers benefitting the diagnosis accuracy of the disease.

## Background

Schizophrenia (SCZ) is one of the most disabling and emotionally devastating illnesses. The global median lifetime morbid risk for schizophrenia is 7.2/1,000 persons [1]. Genetic factors play an important role in the development of schizophrenia. To date, over 1000 genes have been reported to associate with SCZ (http://www.szgene.org/default.asp) and many SNPs have been identified as biomarkers for the disease [2-4]. For example, Kordi-Tamandani et al. showed that that promoter methylation of the CTLA4 gene can increase the risk of SCZ disease [2]. Shayevitz et al. confirmed the gene NOTCH4 as a candidate gene for schizophrenia with genome-wide association studies (GWAS) [3]. Chen et al. stated that three SNPs spanning the MYO5B gene are significantly associated with SCZ: rs4939921, rs1557355 and rs4939924 [4]. Besides genomic data, fMRI is another widely used data modality in SCZ studies [5][6]. To date, many methods have been proposed to integrate multi-types of data in SCZ disease study [7-11]. For example, Chen et al. proposed parallel independent component analysis (paraICA) to identify genomic risk components associated with brain function abnormalities and detected significant biomarkers from both fMRI data and SNP data that are strongly correlated [7]. Parallel ICA is an effective method for the joint analysis of multiple modalities including interconnections between them [8]. Utilizing this method, Meda et al. detected three fMRI components significantly correlated with two distinct gene components in SCZ study [11]. In this study, a novel sparse representation based variable selection (SRVS) method was proposed and applied to an integrative analysis of two types of data: fMRI and SNP, aiming to obtain comprehensive analysis.

Sparse representation including compressive sensing has been widely used in signal/image processing and computational mathematics [12-18]. Candes et al. showed that stable signal can be approximately recovered from incomplete and inaccurate measurements [14]. Wright et al. proposed a sparse representation based clustering (SRC) for face recognition, demonstrating high classification accuracy [15]. In our recent works [16-18], we developed novel classification and feature selection algorithms based on sparse representation theory. We applied those methods to gene expression data analysis [16], to chromosome image classification [18], and to joint analysis of different data modalities (e.g. SNP data and gene expression data) [17], and achieved improved classification accuracies as well as better feature selections.

In applications of sparse representation, The availability of a limited number of samples is an important issue (e.g., feature selection and signal recovery) [19][20][21]. According to compressive sensing theory (e.g., the restricted isometry property (RIP) condition [23][24] for signal recovery), the number of available samples should not be less than the number of signals to be selected/recovered. However, the number of features/variables in genomic data (e.g. SNP data) or medical imaging data (e.g. fMRI data) are usually significantly big than the number of samples. In those cases, the traditional methods for compressive sampling cannot effectively analyse the data.

In a recent work, Li et al. [21] developed a voxel selection algorithm for fMRI data analysis. The method was based on sparse representation and is designed to get a sparse solution when sufficient samples exist. However, it may not handle the small sample problem described above.

In this study, a novel sparse representation based variable selection (SRVS) algorithm was proposed to select relevant biomarkers from big data sets having small sample sizes. The analysis was obtained by using a window based approach, whose size determines the resolution of the variable selection. We first tested the SRVS algorithm on a simulated data set (size of 100 × 1e^6^, with 50 cases and 50 controls), demonstrating the multi-resolution characteristic of the method. Then the algorithm was applied to an integrative analysis of two real data sets: a SNP data set (size of 208 × 759075) and a fMRI data set (size of 208 × 153594). Using the proposed SRVS algorithm, biomarkers for SCZ were identified and validated.

## Methods

### fMRI and SNP data collection

A total of 208 subjects, after signing informed consent, were recruited in the study, including 96 SCZ cases (age: 34 ± 11, 74 males) and 112 healthy controls (age: 32 ± 11, 68 males). Both SNP and fMRI data were collected from each of those 208 subjects. The healthy controls have no history of psychiatric disorders and were free of any medical. SCZ cases met the DSM-IV diagnostic criteria for schizophrenia. After pre-processing, 153594 fMRI voxels and 759075 SNP loci were obtained for the following biomarker selections. Please refer to [22] for detailed description of data collection and pre-processing.

### Generalized sparse model

To combine different data sets for integrative analysis, we consider the following model:

(1) $$y=\left[\alpha_{1}X_{1},\alpha_{2}X_{2}\right]\left[\begin{array}{c} \delta_{1}\\ \delta_{2} \end{array}\right]+\varepsilon =X\delta +\varepsilon$$

where $y\in R^{n\times 1}$ is the phenotype vector of the subjects; matrix $X_{1}\in R^{n\times p_{1}}$ and $X_{2}\in R^{n\times p_{2}}$ represent data sets of different modalities having normalized column vectors (e.g., ${\left||*|\right|}_{2}=1$); $X=\left[\alpha_{1}X_{1},\alpha_{2}X_{2}\right]\in R^{n\times p}$; $\alpha_{1}+\alpha_{2}=1$, and $\alpha_{1},\alpha_{2}>0$ are the weight factors for $X_{1}$ and $X_{2}$ respectively. The measurement error $\varepsilon \in R^{n\times 1}$. We aim to reconstruct the unknown sparse vector $\delta =\left[\begin{array}{c} \delta_{1}\\ \delta_{2} \end{array}\right]\in R^{p\times 1}$ based on  y and  X, where $\delta_{1}\in R^{p_{1}\times 1}$, $\delta_{2}\in R^{p_{2}\times 1}$, and $p_{1}+p_{2}=p$.

It can be proven that when p>35n, the matrix $X\in R^{n\times \text{p}}$ has the difficulty to satisfy the restricted isometry property (RIP) condition [24] for signal recovery. In this work, p=759075+153594=912669 and n=208. Thus p≫35n=7280. To overcome this problem, we propose the SRVS algorithm described as follows.

### SRVS algorithm

To best approximate  y with the model given by Eq. (1), we consider the following $L_{p}$ minimization problem:

(2) $$\text{min}{\left||\delta |\right|}_{p}\;subject\;to\;{\left||y-X\delta |\right|}_{2}\leq \varepsilon$$

where ${\left||*|\right|}_{2}$ represents $L_{p}$ norm; p∈[0,1]. The SRVS Algorithm given below is used to solve the $L_{p}$ minimization problem and select the phenotype relevant column vectors out of  X.

#### Spare representation base variable selection (SRVS) algorithm (http://hongbaocao.weebly.com/software-for-download.html)

1. Initialize $\delta^{\left(0\right)}=0$;

2. For the  l th step, randomly select $X_{l}\in R^{n\times k}$ from $X=\left\{x_{1},\;.\;.\;.\;,\;x_{p}\right\}\in R^{n\times p}$; Mark the indexes of the columns in $X_{l}$ as $I_{l}\in R^{1\times k}$;

3. Solve Eq. (3) to get $\delta_{l}\in R^{k\times 1}$:

(3) $$\text{min}{\left||\delta_{l}|\right|}_{p}\;\text{s}.\text{t}.\;{\left||y-X_{l}\delta_{l}|\right|}_{2}\leq \varepsilon$$

4. Update $\delta^{\left(l\right)}\in R^{p\times 1}$ with $\delta_{l}$: $\delta^{\left(l\right)}\left(I_{l}\right)=\delta^{\left(l-1\right)}\left(I_{l}\right)+\delta_{l}$; where $\delta^{\left(l\right)}\left(I_{l}\right)$ and $\delta^{\left(l-1\right)}\left(I_{l}\right)$ denote the $I_{l}$ th entries in $\delta^{\left(l\right)}$ and $\delta^{\left(l-1\right)}$ respectively;

5. If $||\delta^{\left(l\right)}/l-\delta^{\left(l-1\right)}/{\left(l-1\right)||}_{2}>\alpha$, update l=l+1; go to Step 2.

6. Set $\delta =\delta^{\left(l\right)}/l$. The non-zero entries in  δ correspond to the columns in  X to be selected.

In Step 3, we sought to solve a $L_{0}$ minimization problem using the OMP algorithm [19]. The OMP has been widely used for signal recovery and approximation [18], [26-30].

It can be proven that, by using the SRVS algorithm, one can identify the significant variables with high probabilities. In addition, the SRVS algorithm can be shown convergent for any given  k and  ε, generating an effective solution for the sparse model specified by Eq. (2). In the following section, we discuss the sparsity control issue to determine the number of variables to be selected.

### Sparsity control using  k

**Figure 1:**
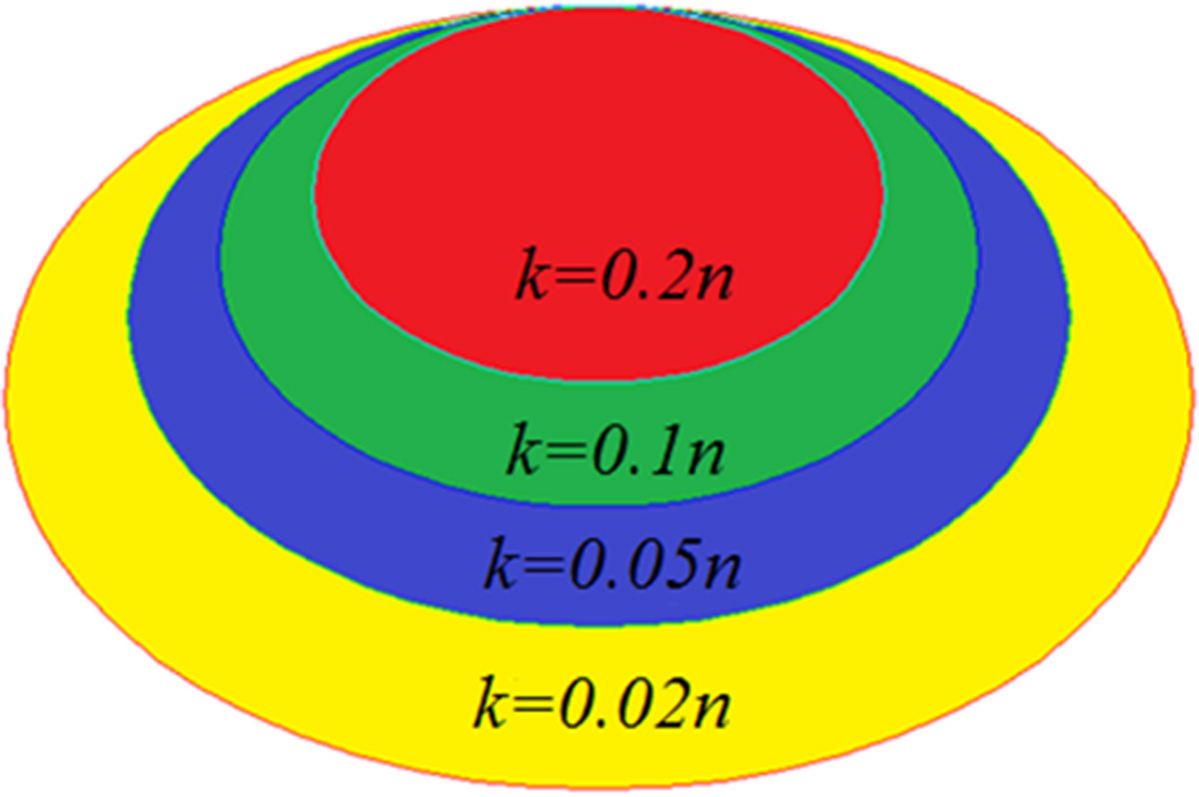
**Diagram for the sparsity control using ** k** in SRVS method**.  p** is the total number of columns/variables**. The results were generated with white noise simulation data set (size 100 × 1e^6^; 50 cases, 50 controls and 1e^6 ^variables).

In Step 2 of the SRVS Algorithm, we exploit Fisher-Yates Shuffling algorithm [31] with a window of length  k to select $X_{l}\in R^{n\times k}$ from $X\in R^{n\times \text{p}}$. The length  k determines the resolution of the SRVS algorithm. When k=p, the number of variables selected will be generally equal to the sample number  n[23]. The smaller the  k, the more the variables selected, and those variables generally include the variables selected with bigger  k, as shown in Figure 1. This multi-resolution property enables us to select different number of variables at different significance levels.

### Further sparsity control using  ε

**Figure 2:**
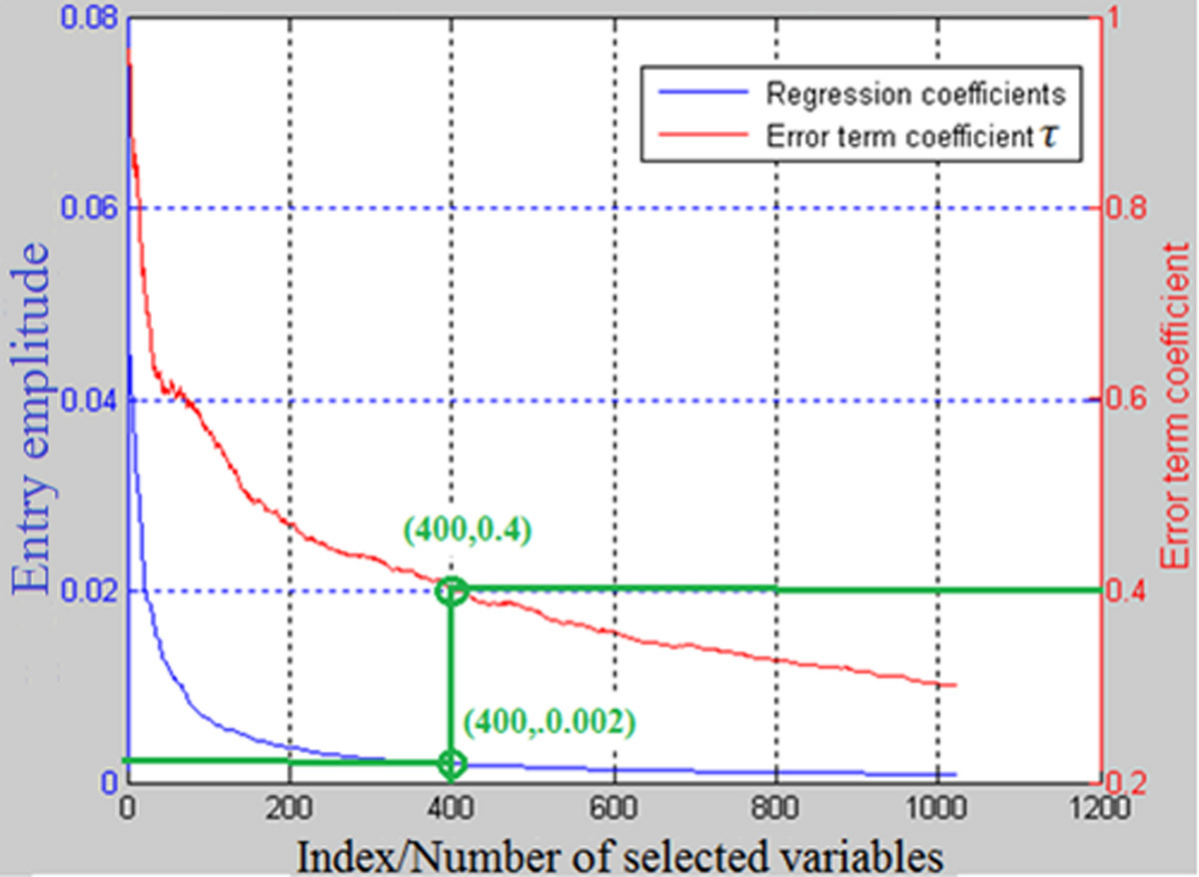
**Diagram for further sparsity control using ** ε. $\varepsilon =\tau {\left||y|\right|}_{2}$; the entries of  δ were sorted in descending order by amplitude. The results were generated with white noise simulation data set of size 100 × 1e^6 ^(50 cases and 50 controls) with $k=0.02\times \text{1}{\text{e}}^{\text{6}}=\text{2}{\text{e}}^{\text{4}}$

The parameter  ε given in Eq. (2) can be used for further sparsity control. The magnitudes of entries of  δ reflect the significance of the corresponding columns of  X[21]. Thus, a threshold can be selected for  δ using cross-validation [32]. Another way to determine a threshold is using the error term  ε (as shown in Figure 2), which reflects the residual of ||y-Xδ||[20]. When ε=0, noises may be involved in the columns selected [20]. In this study, we set $\varepsilon =\tau {\left||y|\right|}_{2}$. From Figure 2, we show that if the first 400 variables with amplitudes larger than 0.002 are selected (i.e. points (400, 0.002) on 'Regression coefficients' curve), it corresponds to the point (400, 0.4) on the 'Error term coefficient' curve; it indicates that with these 400 variables, the error term $\varepsilon =0.4{\left||y|\right|}_{2}$.

### Validation

To validate the variable selected using our proposed SRVS algorithm, we compared our selected SNPs and fMRI voxels with that of previous studies. In addition, we used the selected SNPs and fMRI voxels to identify SCZ patients from healthy controls with the sparse representation based classifier (SRC) [15][18]. Then a leave one out (LOO) cross-validation approach was carried out to evaluate the identification accuracy. We compared the classification results with that of Li et al.'s method [21]. Furthermore, we compared the results of using variables selected from one type of data and that of both types of data. We also studied the influences of selecting different number of variables.

## Result

We applied our SRVS method with the sparse model given by Eq. (1) to an integrative analysis of SNP and fMRI data sets. The results were compared with that of Li et al.'s method under different weighting factors. We also discussed the sparsity control issues using  k and  ε.

### Variable selection with different weight factors

**Figure 3:**
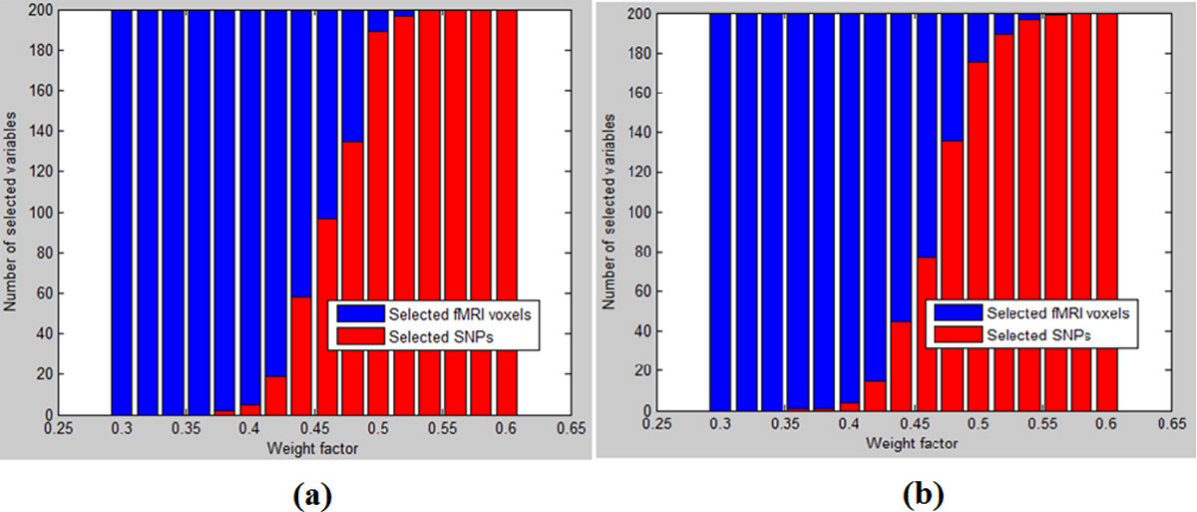
**Variable selection with the sparse model using different methods**. The 'Weight factor' in the plots refers to $\alpha_{1}$ (range of [0.3, 0.6]; step length = 0.02). (a) SRVS method with $L_{0}$ norms (b) Li et al.'s SLR method

Sparse model given by Eq. (1) with different weight factors were solved by our proposed SRVS method and by Li et al.'s method, respectively, as shown in Figure 3. It can be seen that at the two ends ($\alpha_{1}=0.3\;\text{or}\;0.6$), the variables were selected form one type of data.

In each of the 16 trials given by Figure 3, we selected the top 200 biomarkers by our proposed SRVS method and by Li et al.'s method [21]. As shown in Figure 3, the weight factor has similar effects on the variable selection of the two methods. It was interesting to see that even though the number of SNPs was much larger than that of fMRI voxels (759075 vs. 153594), similar number of variables were selected from both data sets when weight factor $\alpha_{1}$ for SNP data set was around 0.5 (0.46 for SRVS method with $L_{0}$ norms, and 0.47 for Li et al.'s method). This suggests that the two data sets may contain similar information for the SCZ case/control study.

### Comparison with Li et al.'s method

**Figure 4:**
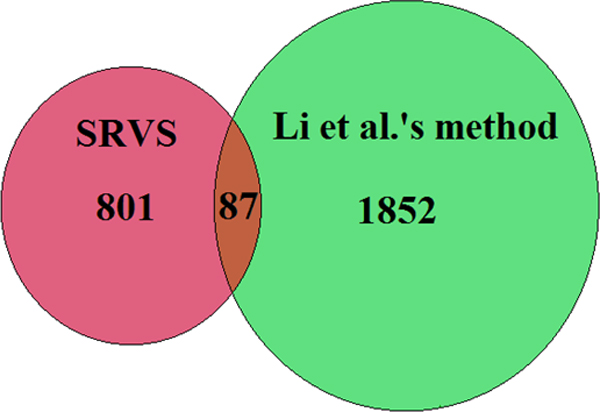
**Comparison of the variables (fMRI voxels/SNPs) selected in the 16 trails by two different methods**.

We selected 200 variables (SNPs and fMRI voxels) in each trial by our proposed SRVS method and by Li's et al.'s method respectively, as shown in Figure 3. However, further study showed that the variables selected by the two methods were significantly different (overlap <10%) (see Figure 4). Thus it was necessary to validate and compare those different groups of variables selected. We first compared the selected SNPs and the corresponding genes with the publicly reported SCZ genes for both methods. Then we compared the brain regions identified using those two methods. In addition, we compared the classification accuracies using the variables selected by our proposed SRVS method and Li et al.'s method.

When compared with the top genes reported (see 'Top 45 SCZ genes' in the Additional file 1). For the 16 trials with the top 200 variables selected in each trial, our proposed SRVS method and Li et al.'s method identified 4 different reported genes, as shown in Table 1. It should be noted that even though both methods can identify gene 'OPCML', they recognized the gene through different SNPs (SRVS is by 'rs3026883' and Li et al.'s method is by 'rs1745939').

**Table 1:** The comparison with the reported first 45 SCZ genes (http://www.szgene.org/default.asp). The Index is the order of the specific gene in the top 45 reported genes list.

**SRVS (**$L_{0}$**)**			Li et al.'s method		
Index	Genes	SNPs	Index	Genes	SNPs

6	PDE4B	rs10846559	1	PRSS16	rs13399561
26	NRG1	rs12097254	11	DAOA	rs16869700
35	PLXNA2	rs4811326	17	RPP21	rs1836942
37	OPCML	rs3026883	37	OPCML	rs1745939

The comparison of selected top SCZ genes by different methods. The Index is the order of the specific gene in the top 45 reported gene list.

To further compare the two methods at different sparsity level, we studied more top variables in each of the 16 trials. To reach this purpose, we set $\varepsilon =0.3y_{2}$ and k=0.05 for SRVS method. For Li's method [21], the number of subjects selected in each run was one tenth of total number of subjects; and we set the threshold  θ = 0.01(please refer to [21] for the meaning of  θ). As a consequence, 500 to 800 variables (SNPs and fMRI voxels) were selected in each trial. In this case, our proposed method selected 20 reported genes. For Li et al.'s method, 14 reported genes were located, and 11 of the top 45 genes were identified by both methods [22]. However, the genes identified by the two methods have <10% overlaps. For the top 50 genes selected by the two methods, there was only one gene, *CSMD1*, was identified by both methods. We listed the top 50 genes and the corresponding SNPs chosen by the two methods in Additional file 2.

**Figure 5:**
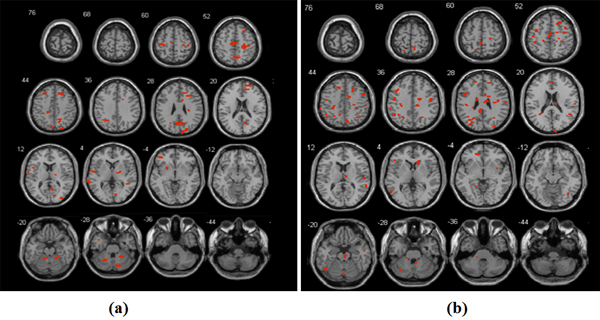
**A comparison of the fMRI voxels selected by using the two different methods**. (a) Voxels selected using SRVS method (b) Voxels selected using Li et al.'s method

When comparing the fMRI voxels selected (follow the approach shown in Figure 3), we showed that the SRVS method were capable of selecting fMRI voxels that were clustered in specific regions, as shown in Figure 5 (a). Those voxels located within a same region will have high correlations with each other. Therefore the results indicate the capability of our proposed SRVS method in selecting significant biomarkers that are highly correlated. Further study showed that the brains regions selected by our proposed SRVS method were mostly reported being associated with SCZ [33-35], including temporal lobe, lateral frontal lobe, occipital lobe, and motor cortex (see Table 2). However, Li et al.'s method tended to select voxels that were scattered over different brain regions (see Figure 5 (b)). Besides, the brain regions selected by those two methods were largely different from each other. Thus we used multivariate classification approach to evaluate the effectiveness of the variables selected by two methods.

**Table 2:** Main brain regions of selected voxels using SRVS method

Brain region	Left(L)/Rigth(R) aal	Selected voxels number
Precuneus	L/R	51
Precentral Gyrus	L/R	35
Sub-Gyral	L/R	32
Middle Frontal Gyrus	L/R	26
Middle Temporal Gyrus	L	20
Cuneus	R	17
Culmen	L/R	16
Paracentral Lobule	L	16
Lentiform Nucleus	L/R	13
Superior Temporal Gyrus	L/R	13
Declive	L/R	13
Cingulate Gyrus	*	13
Postcentral Gyrus	R	9
Medial Frontal Gyrus	R	7
Superior Frontal Gyrus	R	7
Anterior Cingulate	R	7

The main brain regions selected using SRVS method

### Multivariate classification

**Figure 6:**
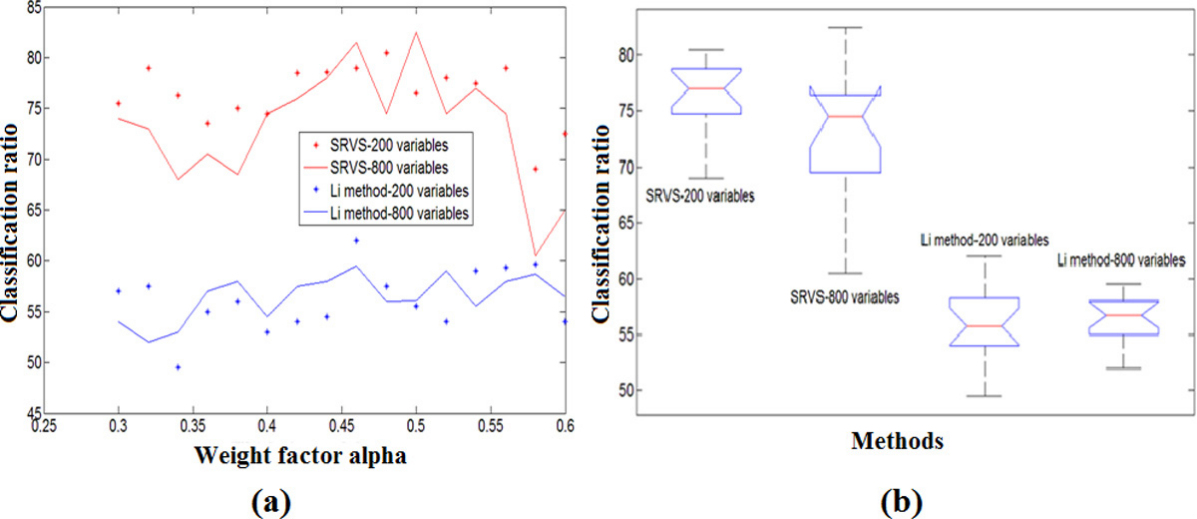
**A comparison of the multivariate classification using two methods**. (a) The classification accuracy of the two methods with different variables selected; (b) The box plots of the classification accuracy. The 'Weight factor' in the plots refers to $\alpha_{1}$, in the range of [0.3, 0.6]; step length = 0.02. (a) CR by using SRVS method (b) CR by using Li et al.'s method

In this study, a LOO cross validation was carried out to evaluate the classification accuracy. In each run of the LOO validation, one sample was used for testing while the rest ones were used for variable selection. Results were presented in Figure 6. We showed that our proposed SRVS algorithm provided significantly higher classification ratios (CRs) ($p-\text{value}\;<{\text{1}}^{\text{e}-\text{11}}$) for both the 200-selected-variable case and the 800-selected-variable case. However, using different number of top selected variables showed no significant differences for neither of the two methods (*p*-value > 0.1).

From Figure 6 (a) we showed that the highest classification accuracy was achieved at the weight factor $\alpha_{1}=0.5$, where around equal sized SNPs and fMRI voxels were selected by the SRVS method. At the two ends ($\alpha_{1}=0.3\;\text{or}\;0.6$), the classification accuracies were relatively lower. This suggests that using biomarkers from both types of data may lead to better identification accuracy.

## Discussion

In this study, we introduced a novel sparse representation based variable selection (SRVS) method, and applied it to an integrative analysis of SNP data and fMRI data. In the case of medical imaging data (e.g. fMRI data) or genomic data (e.g. SNP data), the number of samples tend to be much less than the number of variables (e.g. fMRI voxles; SNP loci). As a consequence, many of those variables are correlated and cannot be identified by traditional sparse signal recovery methods. The proposed SRVS method can identify significant variables with high probability, regardless of the coherence conditions required for exact signal recovery in compressive sensing. For example, significant fMRI voxels functionally correlated (within neighbour brain regions) were identified simultaneously by using our proposed SRVS algorithms (see Figure 5 (a)). This manifests the capability of out proposed SRVS method in handling big data set with small sample sizes.

In addition, the proposed SRVS method can be generalized to integrate multiple data modalities for joint analysis and achieve comprehensive diagnosis. As can be seen from Figure 6 (a), the highest classification accuracy was achieved using approximately equal sized variables from both data sets, suggesting that using biomarkers from both types of data may lead to higher diagnosis accuracy.

Another advantage of the SRVS method is its multiple detection resolutions. By choosing different values of widow length  k one can select different number of variables at different significance level. Furthermore, the error term  ε can be used for further sparsity control of the solution  δ, selecting the most important variables. This multi-resolution characteristic of SRVS provides a flexible variable selection approach for big data sets.

When compared to the previous SCZ studies, our method effectively identified more reported SCZ genes than Li et al.'s method. Furthermore, most of the brain regions identified using our proposed SRVS method are previously reported as SCZ associated brain regions. When using the selected variable to identify SCZ patients from controls, our method generated significantly higher classification ratio than Li et al.'s method (Figure 5 (b), $p-\text{value}\;<{\text{1}}^{\text{e}-\text{11}}$). Those results demonstrated the effectiveness of our method.

## Conclusions

Our proposed SRVS is effective in variable selection for complex disease as SCZ. The biomarkers selected generate better identification accuracy than that of Li et al.'s method. When combining information from fMRI data and SNP data for integrative analysis, higher identification accuracy can be achieved, demonstrating the advantage of the combined analysis.

## Competing interests

The authors declare that they have no competing interests.

## Authors' contributions

HC and YPW designed research. HC designed the algorithm. HC, JD, DL and VC performed data analysis. All authors read and approved the final manuscript.

## Supplementary Material

Additional file 1 **The top 45 schizophrenia genes reported**. Click here for file

Additional file 2 **The top 50 genes and the corresponding SNPs chosen by the two methods proposed SRVS method and Li et al**.'s method. Click here for file

## Declarations

The publication costs for this article were funded by the corresponding author.

This article has been published as part of *BMC Medical Genomics *Volume 6 Supplement 3, 2013: Selected articles from the IEEE International Conference on Bioinformatics and Biomedicine 2012: Medical Genomics. The full contents of the supplement are available online at http://www.biomedcentral.com/bmcmedgenomics/supplements/6/S3.
